# A randomized phase 2 study of neoadjuvant carboplatin and paclitaxel with or without atezolizumab in triple negative breast cancer (TNBC) - NCI 10013

**DOI:** 10.1038/s41523-022-00500-3

**Published:** 2022-12-30

**Authors:** Foluso O. Ademuyiwa, Feng Gao, Cherease R. Street, Ina Chen, Donald W. Northfelt, Robert Wesolowski, Mili Arora, Adam Brufsky, E. Claire Dees, Cesar A. Santa-Maria, Roisin M. Connolly, Jeremy Force, Alvaro Moreno-Aspitia, John M. Herndon, Madelyn Carmody, Sherri R. Davies, Sarah Larson, Kathleen L. Pfaff, Stephanie M. Jones, Jason L. Weirather, Anita Giobbie-Hurder, Scott J. Rodig, Zheng Liu, Ian S. Hagemann, Elad Sharon, William E. Gillanders

**Affiliations:** 1 Washington University School of Medicine, St Louis, MO 63110 USA; 2 Mayo Clinic, Phoenix, AZ 85054 USA; 3 Ohio State University Comprehensive Cancer Center, Columbus, OH 43210 USA; 4 UC Davis Comprehensive Cancer Center, Sacramento, CA 95817 USA; 5 University of Pittsburgh School of Medicine, Pittsburgh, PA 15213 USA; 6 University of North Carolina School of Medicine, Chapel Hill, NC 27514 USA; 7 The Sidney Kimmel Comprehensive Cancer Center at Johns Hopkins, Baltimore, MD 21287 USA; 8 University College Cork, Cork, Ireland; 9 Duke University School of Medicine, Durham, NC 27710 USA; 10 Mayo Clinic, Jacksonville, FL 32224 USA; 11 Cancer Immune Monitoring and Analysis Center, Dana-Farber Cancer Institute, Boston, MA 02215 USA; 12 National Cancer Institute, Bethesda, MD 20892 USA

**Keywords:** Cancer immunotherapy, Breast cancer

## Abstract

Atezolizumab with chemotherapy has shown improved progression-free and overall survival in patients with metastatic PD-L1 positive triple negative breast cancer (TNBC). Atezolizumab with anthracycline- and taxane-based neoadjuvant chemotherapy has also shown increased pathological complete response (pCR) rates in early TNBC. This trial evaluated neoadjuvant carboplatin and paclitaxel with or without atezolizumab in patients with clinical stages II-III TNBC. The co-primary objectives were to evaluate if chemotherapy and atezolizumab increase pCR rate and tumor infiltrating lymphocyte (TIL) percentage compared to chemotherapy alone in the mITT population. Sixty-seven patients (ages 25–78 years; median, 52 years) were randomly assigned – 22 patients to Arm A, and 45 to Arm B. Median follow up was 6.6 months. In the modified intent to treat population (all patients evaluable for the primary endpoints who received at least one dose of combination therapy), the pCR rate was 18.8% (95% CI 4.0–45.6%) in Arm A, and 55.6% (95% CI 40.0–70.4%) in Arm B (estimated treatment difference: 36.8%, 95% CI 8.5–56.6%; *p* = 0.018). Grade 3 or higher treatment-related adverse events occurred in 62.5% of patients in Arm A, and 57.8% of patients in Arm B. One patient in Arm B died from recurrent disease during the follow-up period. TIL percentage increased slightly from baseline to cycle 1 in both Arm A (mean ± SD: 0.6% ± 21.0%) and Arm B (5.7% ± 15.8%) (*p* = 0.36). Patients with pCR had higher median TIL percentages (24.8%) than those with non-pCR (14.2%) (*p* = 0.02). Although subgroup analyses were limited by the small sample size, PD-L1-positive patients treated with chemotherapy and atezolizumab had a pCR rate of 75% (12/16). The addition of atezolizumab to neoadjuvant carboplatin and paclitaxel resulted in a statistically significant and clinically relevant increased pCR rate in patients with clinical stages II and III TNBC. (Funded by National Cancer Institute).

## Introduction

Triple negative breast cancer (TNBC) represents approximately 10–20% of breast cancers and is defined by a lack of expression of the estrogen receptor (ER), progesterone receptor (PR), and human epidermal growth factor receptor 2 (HER2) receptor^1^. TNBC has a higher incidence in young black women and in women with a deleterious mutation in the *BRCA*1 gene^2,3^. Established targeted therapies in breast cancer, such as tamoxifen and trastuzumab, are primarily directed against nuclear or surface receptors. Despite neoadjuvant chemotherapy trials showing that TNBC has higher pathological complete response (pCR) rates to chemotherapy compared to hormone-receptor-positive subtypes^4^, TNBC remains difficult to treat due to chemotherapy resistance, and many patients relapse within 5 years^5^. Patients with TNBC typically have a worse initial prognosis compared to patients with other breast cancer subtypes. Therefore, there is a significant need to develop new treatment strategies for TNBC.

Programmed death ligand 1 (PD-L1) expression is prevalent in many human tumors, and its overexpression has been associated with poor prognosis in patients with several cancers^6–9^. PD-L1 binds to two known inhibitory receptors expressed on activated T cells (PD-1 and B7-1), and PD-L1 expression is sustained in states of chronic stimulation such as cancer^10,11^. Aberrant expression of PD-L1 impedes anti-tumor immunity, resulting in immune evasion^12^. Interruption of the PD-L1/PD-1 and PD-L1/B7.1 pathway reinvigorates tumor-specific T-cell immunity. TNBC has characteristics that may be associated with improved response to immune checkpoint inhibition, including increased mutational complexity^13–16^, higher PD-L1 expression^17–21^, and higher tumor infiltrating lymphocytes (TIL) compared to other breast cancer subtypes^22–25^.

Atezolizumab is a human immunoglobulin (Ig) G1 monoclonal antibody that targets PD-L1 and inhibits its interaction with its receptors, PD-1 and B7-1. Atezolizumab is approved as a single-agent or in combination with other therapy for the treatment of a variety of advanced cancers, including TNBC^26^, urothelial^27^, lung^28–30^, and hepatocellular^31^. In the IMpassion130 study, the addition of atezolizumab to nab-paclitaxel improved progression-free survival (PFS) and a clinically meaningful improvement in overall survival (OS) in patients with metastatic PD-L1-positive TNBC^26,32^. The benefit of immunotherapy in early stage TNBC is being explored in multiple clinical trials. KEYNOTE-522 and IMpassion031 both demonstrated statistically significant improvements in pCR rates when checkpoint inhibitors were added to chemotherapy versus chemotherapy alone^33,34^. However, in the NeoTRIPaPDL1 trial, atezolizumab plus chemotherapy did not significantly improve pCR rates compared with chemotherapy alone in patients with early stage high-risk or locally advanced TNBC^35^. It should be noted that the chemotherapy backbone was carboplatin + nab-paclitaxel, which is not conventional, and the study’s endpoint was event free survival (EFS) and not pCR. Herein, we present the results of the NCI-10013 study (clinicaltrials.gov number, NCT02883062), conducted to evaluate the addition of atezolizumab to non-anthracycline-based neoadjuvant chemotherapy in patients with clinical stages II and III TNBC.

## Results

### Patient characteristics

**Fig. 1:**
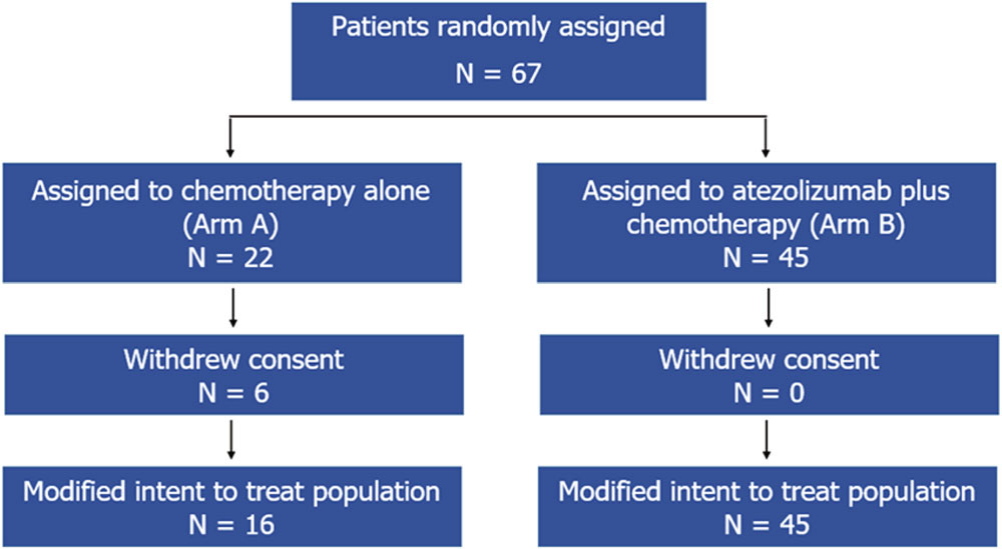
CONSORT diagram. Flow of patients randomly assigned in NCI 10013.

From August 2017 to September 2019, 67 patients from 9 sites were randomly assigned in a 1:2 fashion to either Arm A (22 patients) or Arm B (45 patients). Six patients randomized to Arm A withdrew consent; only 2 of these received protocol therapy and withdrew consent during treatment (Fig. 1). These 2 patients are excluded from the mITT analyses as further data collection did not occur on them due to withdrawal of consent, and they are deemed not evaluable because definitive pathology reports are not available. Median follow up is 6.6 months (range 0.3–15.6 months). The patients’ characteristics are shown in Table 1. Median age is 52 years (range 25–78); 54 years in Arm A and 49 years in Arm B. Overall, forty-three (64.2%) were Caucasian and thirteen (19.4%) were African American. Twenty-five (37.3%) were pre-menopausal. 67.2% and 32.8% had stages II and III disease respectively. Nine (13.4%) had a known germline mutation in either *BRCA1* or *BRCA2*. Genetic testing was according to institutional practices and was not protocol mandated. 54.5% of patients were PD-L1-negative as assessed using the VENTANA PD-L1 (SP142) assay.

**Table 1:** Baseline patient characteristics^a^.

Characteristic	Arm A, *N* = 22 (%)	Arm B, *N* = 45 (%)	*p*-values
Median age	54	49	0.43
Menopausal status			0.07
Premenopausal	5 (22.7)	20 (44.4)	(0.17)^b^
Postmenopausal	14 (63.6)	24 (53.3)	
Unknown	3 (13.6)	1 (2.2)	
ECOG^c^			
0	21 (95.5)	41 (91.1)	>0.99
1	1 (4.5)	4 (8.9)	
Race			0.37
White	16 (72.7)	27 (60.0)	
African American	2 (9.1)	11 (24.4)	
Other	4 (18.2)	7 (15.6)	
Clinical stage			0.78
II	14 (63.6)	31 (68.9)	
III	8 (36.4)	14 (31.1)	
Nodal involvement			0.12
Positive	11 (50.0)	21 (46.7)	(0.60)^b^
Negative	9 (40.1)	24 (53.3)	
Unknown	2 (9.1)	0 (0.0)	
Germline BRCA status			0.40
Wild type	13 (59.1)	34 (75.6)	(0.43)^b^
Mutant	4 (18.2)	5 (11.1)	
Unknown	5 (22.7)	6 (13.3)	
Baseline PD-L1 status			
Negative	5 (22.7)	19 (42.2)	0.015
Positive	4 (18.2)	16 (35.6)	(>0.99)^b^
Unknown	13 (59.1)	10 (22.2)	

^a^Data are shown for the intent to treat population.

^b^Excluding patients with unknown status.

^c^Eastern Cooperative Oncology Group (ECOG) performance status.

### Efficacy

**Fig. 2:**
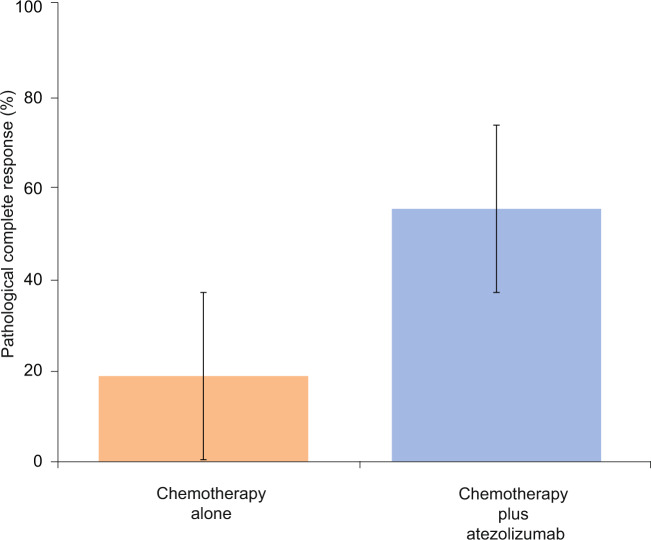
Pathological complete response according to study arm. Arm A (orange bar) represents patients randomly assigned to chemotherapy alone, Arm B (blue bar) represents patients assigned to atezolizumab plus chemotherapy. Error bars represent the 95% confidence interval of pCR rate.

In the mITT population, 3 of 16 patients achieved pCR in Arm A - 18.8% (95% CI 4.0–45.6%), versus 25 of 45 patients in Arm B - 55.6% (95% CI 40.0–70.4%); estimated treatment difference, 36.8 percentage points; *p* value 0.018 (logistic regression, Fig. 2). pCR in those with *BRCA* mutations was 50% (2/4) and 80% (4/5) in Arm A and Arm B, respectively.

### Safety

All patients who received at least 1 cycle of combination chemotherapy were evaluable for toxicity. Treatment delays were observed in 9 patients (40.9%) in Arm A, and 20 (44.4%) in Arm B. Dose reductions occurred in 4 patients (18.1%) in Arm A, and in 6 (13.3%) in Arm B. Grade 3 or higher treatment-related adverse events occurred in 62.5% of patients in Arm A and 57.8% of patients in Arm B (Table 2). The incidence of grade 3/4 anemia was 12.5% in Arm A versus 17.8% in Arm B; neutropenia was 43.8% in Arm A and 33.3% in Arm B. One patient in Arm B had grade 3 adrenal insufficiency. No other immune-related adverse events occurred. Four patients (25%) in Arm A and 10 (22.2%) in Arm B had at least one serious adverse event, defined as any death, life-threatening adverse event, inpatient or prolonged existing hospitalization for ≥24 h, persistent or significant incapacity or substantial disruption of the ability to conduct normal life functions, congenital anomaly/birth defect, or other experience which may jeopardize the subject and may require medical or surgical intervention to prevent one of the afore-mentioned outcomes. No treatment-related deaths occurred, however, one patient in Arm B died from recurrent disease during the follow-up period.

**Table 2:** Adverse events.

Grade 3/4 AEs	Arm A, *N* = 16 (%)	Arm B, *N* = 45 (%)
Adrenal insufficiency	0 (0.0)	1 (2.2)
ALT increase	0 (0.0)	2 (4.40
Anemia	2 (12.5)	8 (17.8)
Anorectal infection	0 (0.0)	1 (2.2)
Arthralgia	1 (6.3)	0 (0.0)
AST increase	0 (0.0)	1 (2.2)
Diarrhea	0 (0.0)	1 (2.2)
Gastroesophageal reflux disease	0 (0.0)	1 (2.2)
Glucose intolerance	0 (0.0)	1 (2.2)
Eye inflammation	1 (6.3)	0 (0.0)
Hypocalcemia	1 (6.3)	0 (0.0)
Hypokalemia	0 (0.0)	1 (2.2)
Hypertension	0 (0.0)	1 (2.2)
Nausea	0 (0.0)	1 (2.2)
Neutropenia	7 (43.8)	15 (33.3)
Thrombocytopenia	1 (6.3)	0 (0.0)
Rash	2 (12.6)	1 (2.2)
Scleral disorder	0 (0.0)	1 (2.2)
Sepsis	0 (0.0)	1 (2.2)
Skin abscess	0 (0.0)	2 (4.4)
Syncope	1 (6.3)	1 (2.2)
Vomiting	0 (0.0)	1 (2.2)
Leucopenia	0 (0.0)	1 (2.2)
**Patients with any SAEs**	**4 (25.0)**	**10 (22.2)**
**SAEs**		
ALT increase	0 (0.0)	1 (2.2)
Anemia	0 (0.0)	1 (2.2)
Anorectal infection	0 (0.0)	1 (2.2)
Arthralgia	1 (6.3)	1 (2.2)
AST increase	0 (0.0)	1 (2.2)
Diarrhea	0 (0.0)	1 (2.2)
Eye inflammation	1 (6.3)	1 (2.2)
Fatigue	0 (0.0)	1 (2.2)
Febrile neutropenia	0 (0.0)	1 (2.2)
Fever	0 (0.0)	1 (2.2)
Fracture	0 (0.0)	1 (2.2)
Hyperglycemia	0 (0.0)	1 (2.2)
Hypocalcemia	1 (6.3)	1 (2.2)
Nausea	0 (0.0)	1 (2.2)
Neutropenia	1 (6.3)	2 (4.4)
Rash	0 (0.0)	1 (2.2)
Sepsis	0 (0.0)	1 (2.2)
Syncope	0 (0.0)	2 (4.4)
Ventricular tachycardia	0 (0.0)	1 (2.2)
Vomiting	0 (0.0)	1 (2.2)

Bold entries highlight SAEs, as separate from all grade 3/4 AEs.

### TIL analyses

**Fig. 3:**
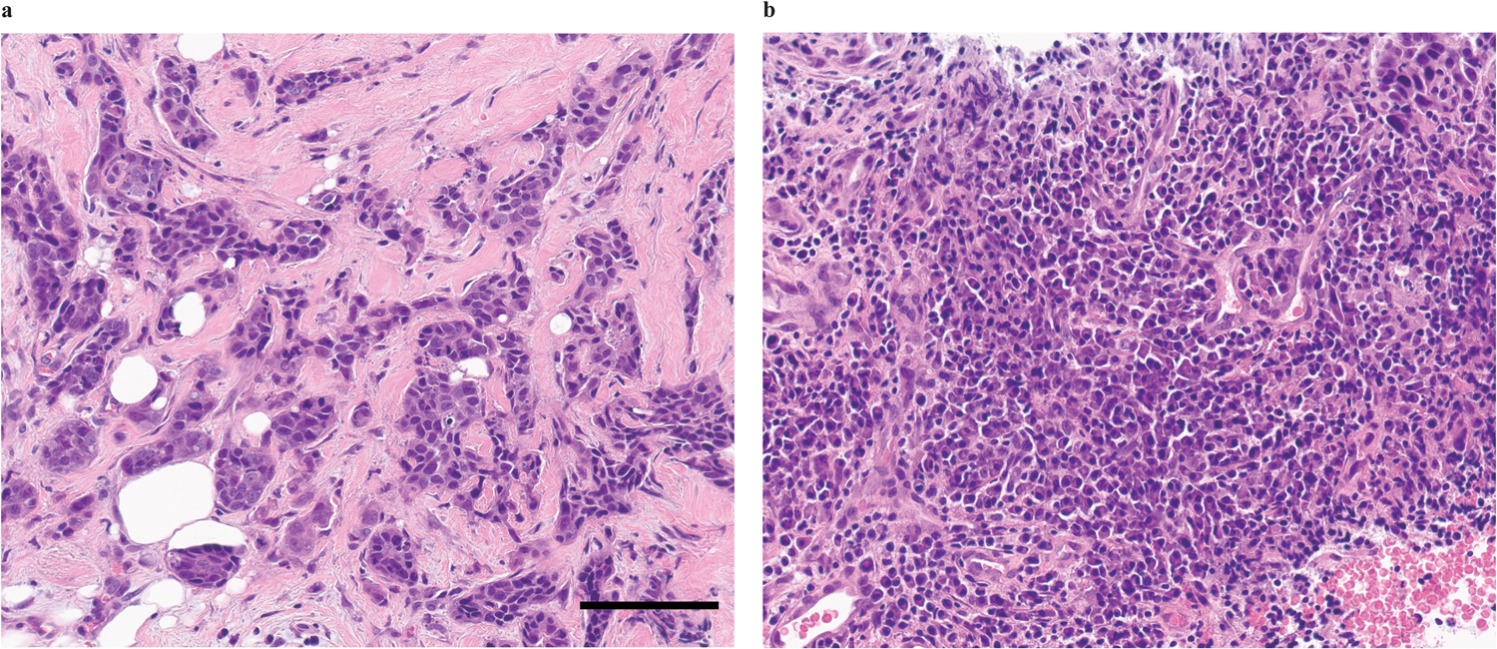
Representative H&E sections demonstrating TIL percentage in breast cancer specimens. **a** Low TIL (5%). **b** high TIL (80%). Scale bar, 100 μm.

TIL percentage is associated with response to adjuvant and neoadjuvant chemotherapy in TNBC^36^. An increase in TIL percentage in response to treatment is also associated with pCR^36^ and response to immune checkpoint inhibition. One of this study’s primary endpoints was to determine whether anti-PD-L1 therapy increases TIL percentage. Forty-eight of 67 participants (71.6%, 13 in Arm A; 35 in Arm B) had tumor specimens with cellularity sufficient for TIL analyses. Other cases could not be assessed for TIL percentage due to the absence of invasive tumor, absence of peritumoral stroma, or due to complete tissue necrosis. We believe that sampling error (rather than response to therapy) is the most likely explanation for the lack of tumor in these samples. Paired tumor samples (taken at baseline, and between days 18–22 following cycle 1 of therapy) for TIL analyses were available on 37 participants (55.2%, 13 in Arm A; 24 in Arm B). Baseline TIL of <50% was observed in 82.6% of all cases (75.0% and 88.5% of pCR and non-pCR cases, respectively). Figure 3 provides examples of representative cases with high and low TIL percentages.

**Fig. 4:**
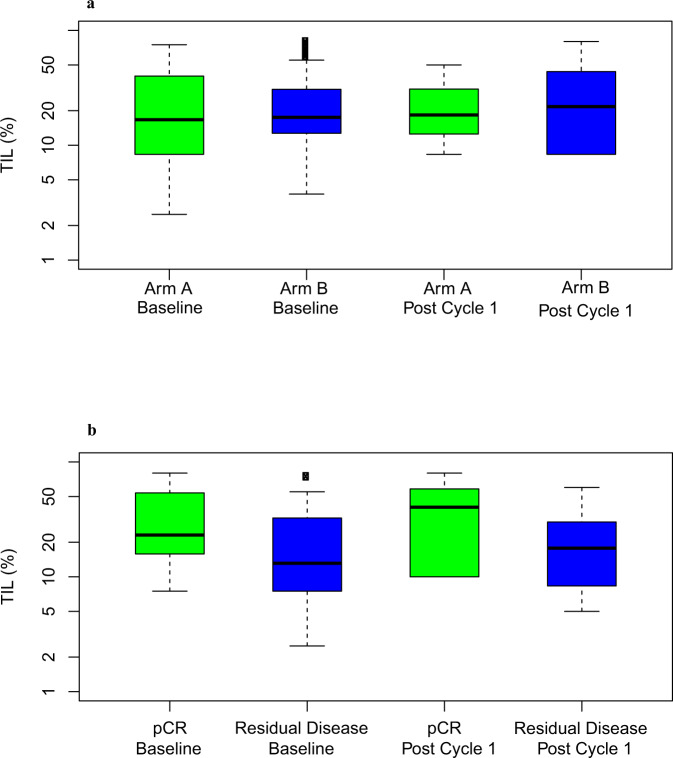
TIL analyses. Boxplots of logarithm transformed TIL (%) by pCR status (**a**) and treatment arm (**b**), where the box stands for the interquartile range, the bar inside a box represents the median, and the whiskers represent the whiskers represent 5^th^ and 95^th^ percentiles.

In Arm A, there was no difference in TIL percentage following treatment when comparing biopsies obtained at baseline and post cycle 1 (GEE; *p* = 0.36): the median TIL percentage at baseline was 16.7% (range 2.5–75.0%), while the median TIL percentage post cycle 1 was 18.3% (range 8.3–50.0%) (estimated mean change in those with paired samples: 0.5%, 95% CI −13.6% - 14.7%). Similarly in Arm B, the administration of atezolizumab did not lead to an increase in TIL percentage (GEE; *p* = 0.72): the median TIL percentage at baseline was 17.5% (range 0–80.0%), while the median TIL percentage post cycle 1 was 21.7% (range 3.3–50.0%) (estimated mean change in those with paired samples: 5.7%, 95% CI −1.4% - 12.9%) (Fig. 4A). The change over time between Arms A and B was also not significant (GEE; *p* = 0.36).

Among the 48 patients with TIL results, pCR rates in Arms A and B were 7.7% and 54.3%, respectively. Evaluating all TIL data by using methods for repeated measurements, patients who achieved pCR had higher median TIL percentages (24.8%) than those with residual disease (14.2%) (GEE; *p* = 0.02) (Fig. 4B). Evaluating TIL at baseline only, there was a trend toward higher median TIL percentages in patients who achieved pCR (23.1%) versus those with residual disease (13.1%) (GEE; *p* = 0.08).

### PD-L1 analyses

To assess PD-L1 expression two assays were utilized. First, PD-L1 expression was evaluated using the VENTANA PD-L1 (SP142) assay^37^. This assay was performed by a CLIA-certified laboratory. A multiplex immunofluorescence assay (mIF) panel was also performed including PD-1, PD-L1, Pan Cytokeratin, CD8, and FOXP3.

**Fig. 5:**
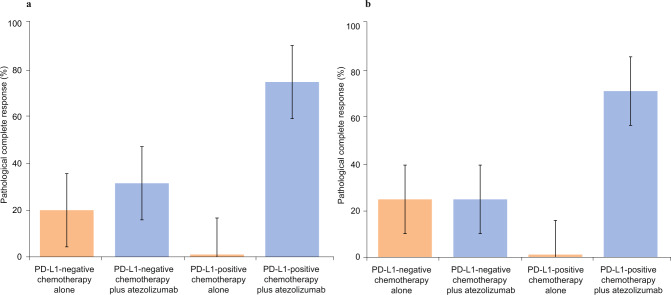
Pathological complete response by PD-L1 status. **a** PD-L1 status assessed by VENTANA PD-L1 (SP142) assay in patients treated with chemotherapy alone and chemotherapy plus atezolizumab. Orange areas represent Arm A chemotherapy only. Blue areas represent Arm B chemotherapy plus atezolizumab. **b** PD-L1 status assessed by multiplex immunofluorescence in patients treated with chemotherapy alone and chemotherapy plus atezolizumab. PD-L1-positive status represents the percent positive cells on inflammatory cells (PD-L1-positive cytokeratin-negative or CD8-positive cells), divided by the total number of cytokeratin-negative and CD8 positive cells. Orange areas represent Arm A chemotherapy only. Blue areas represent Arm B chemotherapy plus atezolizumab. Error bars represent standard error of pCR rate.

When we assessed PD-L1 status using the VENTANA PD-L1 (SP142) assay, we observed numerically improved response rates with chemotherapy plus atezolizumab in both PD-L1-negative and PD-L1-positive patients. The pCR rate was 20% (1/5) vs. 31.6% (6/19) in PD-L1-negative patients, and 0% (0/4) vs 75% (12/16) in PD-L1-positive patients (logistic regression; *p* > 0.99 and *p* = 0.01, respectively) in arm A vs. arm B, respectively. (Fig. 5A).

When we assessed PD-L1 status using mIF analyses, we observed similar results. For these analyses, we used clone E1L3N to assess PD-L1 expression. PD-L1 expression was quantified based on count density (cells/mm^2^). For data analysis, we used a cutoff of ≥1% cytokeratin-negative, PD-L1-positive cells. This is the best approximation of the VENTANA PD-L1 (SP142) assay cutoff of ≥1% immune cells. Based on this analysis, the pCR rate was 25% (1/4) vs 25% (1/4) in PD-L1-negative patients, and 0% (0/3) vs 72% (13/18) in PD-L1-positive patients (logistic regression; *p* > 0.99 and *p* = 0.04, respectively) (Fig. 5B).

Additional TIL correlative analyses were conducted to compare TIL percentages by PD-L1 status. We compared baseline TIL percentage in PD-L1-negative and PD-L1-positive patients by SP142. We found that PD-L1-positive patients have a statistically significant higher TIL percentage. The median TIL percentage was 12.5% in PD-L1-negative patients and 33.1% in PD-L1-positive patients (GEE; *p* = 0.006). In PD-L1-negative patients, there was a statistically significant difference in TIL percentage between patients with residual disease and pCR (median TIL 8.3% vs. 20.2%; *p* = 0.03; GEE). In PD-L1-positive patients, there was no statistically significant difference in TIL percentage between patients with residual disease and pCR (median TIL 33.8% vs. 28.8%; *p* = 0.66; GEE) (Table 3).

**Table 3:** Baseline TIL (%) by PD-L1 and pCR status (Arms A and B).

PD-L1 status	Covariate	Statistics	Residual disease (*N* = 24)	pCR (*N* = 17)	*P*-value*	*P*-value**
Negative	TIL%	*N*	17	6	0.037	0.007
		Mean	13.48	30.28		
		Median	8.75	20.21		
Positive	TIL%	*N*	7	11	0.662	
		Mean	40.06	40.95		
		Median	33.75	28.75		

*p-value for non-pCR vs. pCR groups, stratified by PD-L1 status; **p-value for PD-L1-negative vs. PD-L1-positive.

### Multiplex Immunofluorescence

Multiplex immunofluorescence (mIF) data were analyzed using the mean cell density (cells/mm^2^). After performing PD-L1 (E1L3N) analysis, we assessed the remaining markers PD-1, Cytokeratin (CTYOK), CD8, and Foxhead Box P3 (FOXP3). The data demonstrated no statistically significant difference between the abundance of T- cells (CD8+), CD8+PD-1+ cells, and T-regulatory cells (FOXP3) between patients with a pathological complete response and non-pathological complete response in Arm B, *p* = 0.25, *p* = 0.33, *p* = 0.48 (generalized linear regression with negative binomial distribution) respectively as shown in Supplementary Fig. 1.

### Subgroup analyses

We performed analyses to evaluate for any potential association between menopausal status and pCR, as well as nodal status and pCR. In the pre-menopausal patients, the pCR rates in Arms A and B were 66.7% (2/3) and 65% (13/20), respectively, and the difference was not significant (logistic regression; *p* = 0.95). In the post-menopausal patients, the pCR rates in Arms A and B were 8.3% (1/12) and 45.8% (11/24), respectively, and there was a trend toward significant difference (logistic regression; *p* = 0.059) (Supplementary Fig. 2). In the patients with negative lymph node at baseline, the pCR rates in Arms A and B were 25% (2/8) and 54.2% (13/24), respectively, and the difference was not significant (logistic regression; *p* = 0.21) In the patients with positive lymph node at baseline, the pCR rates in Arms A and B were 12.5% (1/8) and 57.1% (12/21), respectively, and there was a trend toward significant difference (logistic regression; *p* = 0.072) (Supplementary Fig. 3).

## Discussion

In this randomized open label phase 2 trial, patients with previously untreated early stage TNBC who received neoadjuvant atezolizumab plus chemotherapy achieved a statistically significantly higher pCR rate than those treated with chemotherapy alone. We used an anthracycline-free platinum-based neoadjuvant backbone consisting of carboplatin and paclitaxel. Platinum-taxane chemotherapy with PD-L1 and/or PD-1 inhibition has been shown to be safe in trials in different tumor types. Anthracyclines are associated with increased cardiac toxicity, myelodysplastic syndromes, and treatment-related leukemia^38,39^. Previous studies have shown that non-anthracycline based regimens in patients with TNBC achieve similar pCR rates as anthracycline plus taxane based regimens^40–43^. Although cross-trial comparisons may be confounded by differences in trial characteristics, the high pCR rate observed in the anthracycline-free experimental arm of this study is similar to rates observed in other neoadjuvant trials utilizing anthracyclines, taxanes, and carboplatin in TNBC^44,45^. Therefore, further studies evaluating non-anthracycline-based chemoimmunotherapy regimens as an alternative strategy in early TNBC are warranted.

During the conduct of this study, several other neoadjuvant trials with immunotherapy in early TNBC were presented. The results of the present trial are similar to results from KEYNOTE-522, which evaluated checkpoint inhibition with pembrolizumab and anthracycline-taxane and carboplatin based neoadjuvant chemotherapy in TNBC^33^ KEYNOTE-522 randomly assigned patients with early stage TNBC to receive neoadjuvant therapy with four cycles of carboplatin and paclitaxel, followed by four cycles of doxorubicin or epirubicin with or without pembrolizumab. The pCR rate in the pembrolizumab group was 63% (95% CI: 59–66%) and 56% (95% CI: 51–61%) in the placebo group^46^ (updated results from FDA press release dated 7/26/21). Similarly, IMpassion031 demonstrated a higher pCR rate in patients with TNBC who received neoadjuvant atezolizumab with nab-paclitaxel followed by ddAC (58%, 95% CI 50–65%) versus those randomized to placebo with chemotherapy (41%, 95% CI 34–49%) (*p* = 0.0044)^34^. Conversely, the phase 3 NeoTRIPaPDL1 study evaluating atezolizumab with carboplatin and nab-paclitaxel did not lead to an increase in pCR rates compared with carboplatin and nab-paclitaxel alone (43.5% with atezolizumab versus 40.8% with chemotherapy alone, OR 1.11 [95% CI 0.69–1.79])^35^. pCR was a secondary endpoint in NeoTRIPaPDL1 and was reported prior to event free survival results, the primary trial endpoint. A notable difference in our trial and NeoTRIPaPDL1, is that NeoTRIPaPDL1 accrued patients who appeared to be higher risk (87% clinically node positive) than the current trial population (48% clinically node positive), although the subgroup analysis in Supplementary Fig. 3 demonstrates no significant difference in pCR rates between lymph node-positive and negative patients. In addition, the carboplatin was administered at a higher 3-weekly dosing schedule in this trial, versus a lower weekly schedule in NeoTRIPaPDL1.

The pCR rate in the chemotherapy arm in this study is lower than that reported in the literature for other recent studies of platinum-based anthracycline-free neoadjuvant chemotherapy for early TNBC. After careful review of the literature, we identified three recent trials in patients with TNBC who were treated with carboplatin and taxane-based neoadjuvant chemotherapy regimens. These studies provide additional information about the expected pCR rate for the patients treated with chemotherapy alone in this study. Sharma et al. reported the results of two independent and separate prospective cohorts of patients with stages I-III TNBC treated with six cycles of carboplatin and docetaxel (NCT02302742 and NCT01560663)^40^. The pCR rate was 55% (95% CI: 48–62%) in 183 evaluable patients^40^. Additionally, Ademuyiwa et al. reported the results of a prospective single institution cohort that included 127 patients with clinical stages II-III TNBC who received also received six cycles of docetaxel and carboplatin (NCT02124902). This study had a pCR rate of 45.7% (95% CI 36.9–54.7%)^47^. The NeoTRIPaPDL1 study discussed above reported a pCR rate of 40.8% in the chemotherapy alone arm. These studies demonstrate a range of pCR rates from 40-56%. There are significant differences between the trials reviewed and the current trial being reported here. One of the most important differences is the dose of chemotherapy, and the number of cycles administered. A second important difference is the disease stage of the patients included in the trials. It is plausible that the pCR rate associated with neoadjuvant carboplatin plus taxane chemotherapy regimens is dose dependent. The comparative underperformance of this trial may be due to the less aggressive chemotherapy regimen. It is notable that the current trial used four cycles of carboplatin while similar trials used six cycles of carboplatin (NCT02302742, NCT01560663, and NCT02124902). Our trial also enrolled stage II-III patients, whereas the trials reported by Sharma et al. enrolled stage I-III patients (NCT02302742, NCT01560663). Based on the small number of evaluable patients in our chemotherapy alone arm and these other considerations, we are not surprised that the pCR rate in the neoadjuvant chemotherapy alone arm was <40%.

Although pCR is a surrogate for long term outcomes in TNBC^4,48^, the benefit of immunotherapy in metastatic TNBC is modest in response endpoints but more substantial in longer term clinical outcomes. In the ITT population in IMpassion130, the difference in objective response rates between those who received atezolizumab and those who did not was only 10.1%^26^. In the PD-L1-positive population, the absolute median PFS improvement was 2.2 months with atezolizumab and chemotherapy relative to chemotherapy alone, whereas final overall survival analysis in the PD-L1 positive patients showed a numerical improvement in median OS of 7.5 months^26,32,49^. This phenomenon has also been observed in other advanced cancers. For example, in non-small cell lung cancer KEYNOTE-189 showed an absolute median PFS improvement of 3.9 months with pembrolizumab added chemotherapy relative to chemotherapy alone, while the median OS was not reached in the pembrolizumab group and was 11.3 months in the chemotherapy alone group^50^. Therefore, it is possible that follow up data from this present trial and NeoTRIPaPDL1 may translate into beneficial long term efficacy.

The addition of atezolizumab to platinum-based chemotherapy was not associated with an increase in adverse events or dose modifications. There were very few grade 3 and 4 events, with the exception of anemia and neutropenia which are both consistent with the well-established safety profile of platinum-based neoadjuvant chemotherapy. The incidence of serious adverse events was also similar in both study groups and no new safety signals were identified. Immune-related adverse events can be permanent, and associated with the requirement for long term hormone replacement therapy^51,52^. During this limited exposure to atezolizumab, adrenal insufficiency was the only immune-related adverse event observed in one patient in the atezolizumab arm.

We conducted analyses of the immune biomarker PD-L1. In subgroup analyses using two different strategies to assess PD-L1 status, we observed numerically improved response rates with chemotherapy plus atezolizumab in both PD-L1-negative and PD-L1-positive patients. The improved response rates were statistically significant for PD-L1-positive patients. Of particular note, we observed that PD-L1-positive patients treated with chemotherapy plus atezolizumab had very high response rates (75% pCR rate). Our findings are best interpreted in the context of several recently reported studies. First, in the neoadjuvant setting, several studies have demonstrated that PD-L1-positive patients have improved response rates to both chemotherapy alone, and chemotherapy plus atezolizumab. Second, in the neoadjuvant setting, both PD-L1-positive and PD-L1-negative patients appear to derive a benefit from anti-PD-L1 therapy. By contrast, in patients with metastatic TNBC, PD-L1-positive patients have a trend towards shorter PFS and overall survival compared to PD-L1-negative patients when treated with chemotherapy alone, and only PD-L1-positive patients benefit from anti-PD-L1 therapy^32,33^. Reasons for the differences in the predictive and prognostic impact of PD-L1 status according to disease stage are unclear, but may represent differences in anti-tumor immunity and immune microenvironment by stage.

In the analysis of the remaining mIF markers we compared the mean cell densities (cells/mm^2^) of T- cells (CD8+), CD8+PD-1+ cells, and T-regulatory cells (FOXP3) cell populations between patients with a pathological complete response and non-pathological complete response in Arm B. There was no statistically significant difference between the abundance of these cell populations in our subgroup analysis.

Immune cells are a defining factor in tumor progression and metastasis, and during phases of the immunoediting process, immune cells may serve different roles in tumor progression^53,54^. Understanding the relationship between the TNBC tumor immune microenvironment and benefit of immunotherapy may help inform which combination strategies are most appropriate for which group of patients with TNBC. Consistent with prior studies^25,55,56^, we found that patients who achieved pCR had higher TIL percentages than patients with residual disease. Although atezolizumab did not significantly increase TIL percentage in general, there was a trend of a larger difference in TIL percentages in pCR patients (almost all from Arm B) versus non-pCR patients after cycle 1 versus at baseline suggesting that atezolizumab may have increased TIL in responding patients. More studies will need to clarify if amplifying TIL with checkpoint inhibitors will lead to clinical benefit in patients with TNBC.

Strengths of this study include the high proportion of African Americans accrued, which is likely reflective of the sites included and the commitment of the investigators to accrue minorities. Furthermore, this was a multicenter collaborative study which simultaneously served as a platform for biomarker analyses. Study limitations include the significant number of patients in the control arm who withdrew consent due to open label design of this study. The selective dropout from Arm A does not appear to have introduced any selection bias as the baseline patient characteristics are equally distributed between the Arms as documented in Table 1.

In conclusion, among patients with early stage TNBC, the proportion who achieved pCR was statistically significantly higher among those who received neoadjuvant atezolizumab plus chemotherapy than among those who received neoadjuvant chemotherapy alone. Non-anthracycline-based chemotherapy plus immunotherapy may be an alternative option for the management of patients with early stage TNBC. The long-term results of this trial and several other randomized trials will together shape the landscape of checkpoint inhibition in the management of early stage TNBC.

## Methods

### Patient eligibility

Eligible women had newly diagnosed untreated TNBC, defined as ER and PR < Allred score of 3 or ≤5% positive staining cells in the invasive component of the tumor. HER2 negative disease was defined as negative by FISH or IHC staining 0 or 1+ according to the American Society of Clinical Oncology and the College of American Pathologists guidelines^57^. Patients had clinical stage T2-T4c, any N, M0 primary tumor by AJCC 7^th^ edition clinical staging. Other key eligibility requirements were age ≥18 years, Eastern Cooperative Oncology Group (ECOG) performance status of ≤2, adequate organ function, and willingness and ability to provide informed consent. Patients with contralateral breast cancer, a history of autoimmune disease, uncontrolled intercurrent illness, or treatment with immunosuppressive medications were excluded.

### Study design

The study was approved by the National Cancer Institute (NCI) Central Institutional Review

**Fig. 6:**
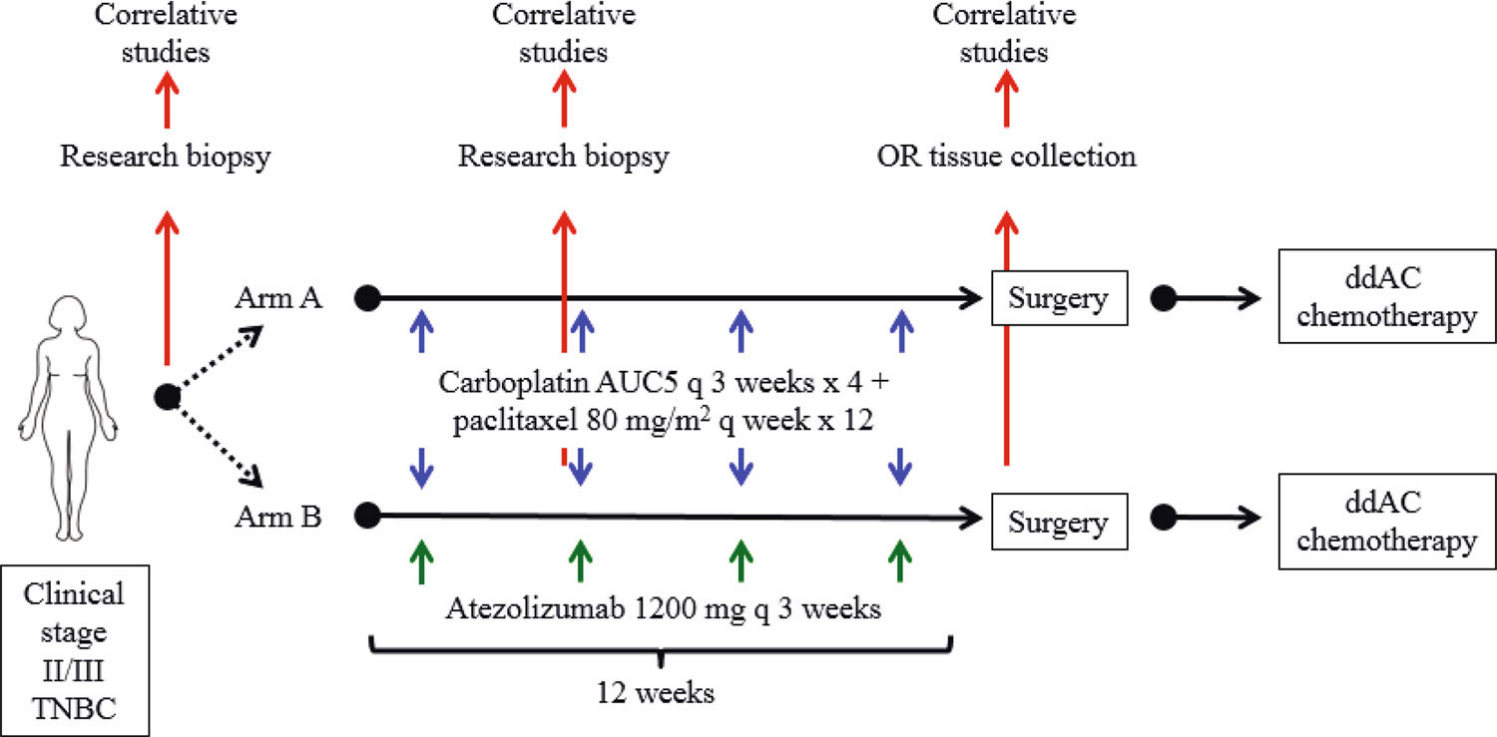
Clinical trial schema outlining treatment arm, randomization, collection of biopsies, and adjuvant treatment. AUC area under the curve, dd dose dense.

Board (IRB) and each of the following independent ethics committees: University Health Network Princess Margaret Cancer Center Research Ethics Board, City of Hope Comprehensive Cancer Center IRB, Yale School of Medicine IRB and Human Investigations Committee, Dana- Farber Cancer Institute single IRB, The Sidney Kimmel Comprehensive Cancer Center at Johns Hopkins Clinical Research Review Committee, The Mayo Clinic IRB, Rutgers Cancer Institute of New Jersey IRB, The Ohio State University Office of Responsible Research Practices and IRB, University of Pittsburgh Cancer Institute Research Ethics Advisory Board and IRB, University of Texas MD Anderson Cancer Center IRB, Duke University Health System, University of California Davis Comprehensive Cancer Center IRB, and Washington University IRB. The study was performed in accordance with the International Conference on Harmonization guidelines concerning Good Clinical Practice and the Declaration of Helsinki. ClinicalTrials.gov number is NCT02883062 and registration date was August 29, 2016. All patients provided written informed consent. Patients were recruited from nine sites within the NCI Experimental Therapeutics Clinical Trials Network (ETCTN) to this open-label phase 2 study (Washington University School of Medicine, Mayo Clinic Arizona, Mayo Clinic Florida, Ohio State University, UC Davis, University of Pittsburgh, University of North Carolina, Duke University, and Johns Hopkins University). The study design is shown in Fig. 6. All eligible patients were randomly assigned, in a 1:2 ratio to the control arm with carboplatin AUC5 every 3 weeks ×4 cycles plus paclitaxel 80 mg/m^2^ every week ×12 weeks (Arm A), or the investigational arm with carboplatin AUC5 every 3 weeks ×4 cycles plus paclitaxel 80 mg/m^2^ every week ×12 weeks plus atezolizumab 1200 mg every 3 weeks ×4 cycles (Arm B). Atezolizumab was provided by the Cancer Therapy Evaluation Program (CTEP) as an investigational drug. Randomization was centralized using the Interactive Web Response System (IWRS). Patients were stratified according to clinical stage (II versus III). Definitive surgery was 3–6 weeks following the completion of neoadjuvant chemotherapy. All patients received dose-dense AC (ddAC) administered in the adjuvant setting as per routine care. ddAC consists of doxorubicin 60 mg/m^2^ on day 1 of each 2-week cycle plus cyclophosphamide 600 mg/m^2^ on day 1 of each 2-week cycle ×4 cycles. Patients who underwent breast conservation therapy (BCT) received adjuvant radiation according to institutional practices. Patients were followed for one year after removal from study or until death, whichever occurred first.

Research tumor biopsies for correlative studies were obtained at baseline prior to chemotherapy, prior to cycle 2, and at time of definitive surgery following neoadjuvant chemotherapy in those patients with residual disease. Blood for correlative studies was also collected (optional) at the same time points.

### TIL percentage assessment

TIL were quantified according to the method recommended by the International Immuno-Oncology Biomarker Working Group on Breast Cancer^58^. Two subspecialized breast pathologists (IH, ZL) independently reviewed whole slide scans (blinded to each other, treatment status, and time point) and documented the percentage of TIL infiltration in tumor-adjacent stroma, in increments of 5%. Discrepancies of >10% points were adjudicated in a consensus conference setting. The mean of the two observations, or the consensus determination if applicable, was used for analysis.

### PD-L1 immunohistochemistry

Evaluation of PD-L1 expression was performed at HistoGeneX (now CellCarta NV) laboratory located in Naperville, IL USA using the VENTANA SP142 IHC assay performed on the Ventana BenchMark ULTRA Instrument (Ventana Medical Systems, Tucson AZ) using the Optiview DAB IHC Detection system. Immune infiltrate staining is scored as the area of PD-L1 positive immune infiltrating cells as a percentage of total tumor area. Immune infiltrate cells include immune cells showing PD-L1 staining (punctate staining as well as circumferential staining) that include lymphocytes, macrophages, and cells with dendritic and reticular morphology. Tumor area is the area occupied by tumor cells as well as their associated intratumoral and contiguous peritumoral desmoplastic stroma. Given the fact the immune cells are present not only within the stroma, but also are seen as single cells or diffuse spread within the tumor cells, tumor area is chosen as the denominator. The fraction of viable Tumor Cells (TC) percentage that express PD-L1 (discernible membrane staining of any intensity) can be scored. Cytoplasmic staining is not included in the scoring. The immune cell (IC) score is a relative area estimate percent of tumor area that is covered by PD-L1-positive ICs. For the purposes of this study a cutoff of ≥1% IC was considered positive.

### Multiplex immunofluorescence

Multiplex immunofluorescence (mIF) was performed using a Bond RX fully automated stainer (Leica Biosystems, Inc.) on 5-µm-thick FFPE (formalin-fixed, paraffin-embedded) whole tissue sections. Slides were baked for 3 h at 60 °C, loaded onto the Bond RX, deparaffinized (BOND DeWax Solution, Leica Biosystems) and rehydrated with a graded ethanol series. Sequential rounds of antigen retrieval, primary antibody incubation, and Opal^TM^ Fluorophore (Akoya Biosciences) incubation were carried out as detailed in Table below, followed by staining with nuclear counterstain/4′,6-diamidino-2-phenylindole (DAPI). All slides were scanned at 20x resolution using a Vectra Polaris imaging platform (Akoya Biosciences). Regions of Interest (ROIs) were selected for each sample with pathologist supervision (SJR). Images were segmented, phenotyped and scored for each individual marker using InForm Image Analysis software (Akoya Biosciences). A custom script was used to extract the density and percentage of cells which are positive for relevant biomarkers in specific tissue regions (tumor and tumor-stroma interface).

**Table:** 

Target	Antibody clone #	Antigen retrieval	Primary antibody dilution	Opal fluorophore
FOXP3	D608R (Cell Signaling)	ER 2, 40 min	1:100	570
PD-L1	E1L3N (Cell Signaling)	ER 1, 20 min	1:300	520
Cytokeratin	AE1/AE3 (Agilent)	ER 1, 20 min	1:100	690
PD-1	EPR4877 (Abcam)	ER 1, 20 min	1:300	620
CD8	4B11 (Leica)	ER 1, 20 min	1:200	480

### Statistical analyses

The co-primary endpoints are pCR rate, and differences in TIL percentage from baseline to after initiation of therapy (day 18–22). pCR is defined as absence of residual invasive cancer in the surgical breast tissue and lymph node specimens. Other objectives included assessing the safety of the combination of carboplatin plus paclitaxel plus atezolizumab, evaluating potential biomarkers of response to the combination, evaluating the impact of the combination on immune response, and evaluating the impact of the combination on overall survival (OS) and disease-free survival (DFS).

The study was designed to enroll 20 patients to Arm A and 40 patients to Arm B to provide 80% power at 1-sided alpha = 0.10 to detect a minimum of 15% difference in TIL percentage, and 29% improvement (69% versus 40%) in pCR. pCR results are presented in the modified intention-to-treat (mITT) population, which includes all randomized patients who were evaluable for the primary endpoint and received at least one dose of combination therapy. Patient demographics and clinical characteristics were summarized using counts and frequencies for categorical variables, or means and standard deviations for continuous variables. The distributions of these baseline factors across arms were compared using two-sample t-test, or Fisher’s exact test as appropriate. The 95% exact binomial confidence limits for pCR was calculated in each arm. The difference in pCR rates between two arms (overall and by subgroups) was described using Miettinen-Nurminen confidence intervals and compared by univariate Firth logistic regression models. TIL percentage was summarized as means, medians, and standard deviations at each time point. The over-time changes and between-group differences in TIL percentages were assessed using generalized estimating equation (GEE) which is less sensitive to the assumption of normality distribution. Natural logarithm transformation was also performed in TIL percentages to reduce its right-skewing. All the statistical analyses were performed using SAS 9.4 (SAS Institutes, Cary, NC).

### Reporting summary

Further information on research design is available in the Nature Research Reporting Summary linked to this article.

## Supplementary information

Supplementary Material

Reporting Summary

## Data Availability

The clinical and other assay data generated in this study will be made publicly available in the NCI Clinical Trials Data Commons (CTDC) and Cancer Data Service (CDS) components of the NCI’s Cancer Research Data Commons (CRDC). Access to these repositories is available at the links below: https://datacommons.cancer.gov/repository/clinical-trial-data-commonshttps://datacommons.cancer.gov/repository/cancer-data-service#:~:text=The%20Cancer%20Data%20Service%20(CDS,generated%20by%20NCI%20funded%20programs These repositories are being prepared to receive the data and data-sharing logistics are being coordinated.
