# Supplementary material for: A randomized phase 2 study of neoadjuvant carboplatin and paclitaxel with or without atezolizumab in triple negative breast cancer (TNBC) - NCI 10013
Source: NPJ Breast Cancer. 2022 Dec 30;8:134. doi: 10.1038/s41523-022-00500-3 (PMC9803651; doi:10.1038/s41523-022-00500-3)
Supplement: Supplementary file 1 — Supplementary Material [file 41523_2022_500_MOESM1_ESM.pdf]

SUPPLEMENTARY FIGURES

Supplementary Figure 1. Multiplex immunofluorescence markers by pathological complete response in patients treated with chemotherapy plus atezolizumab.

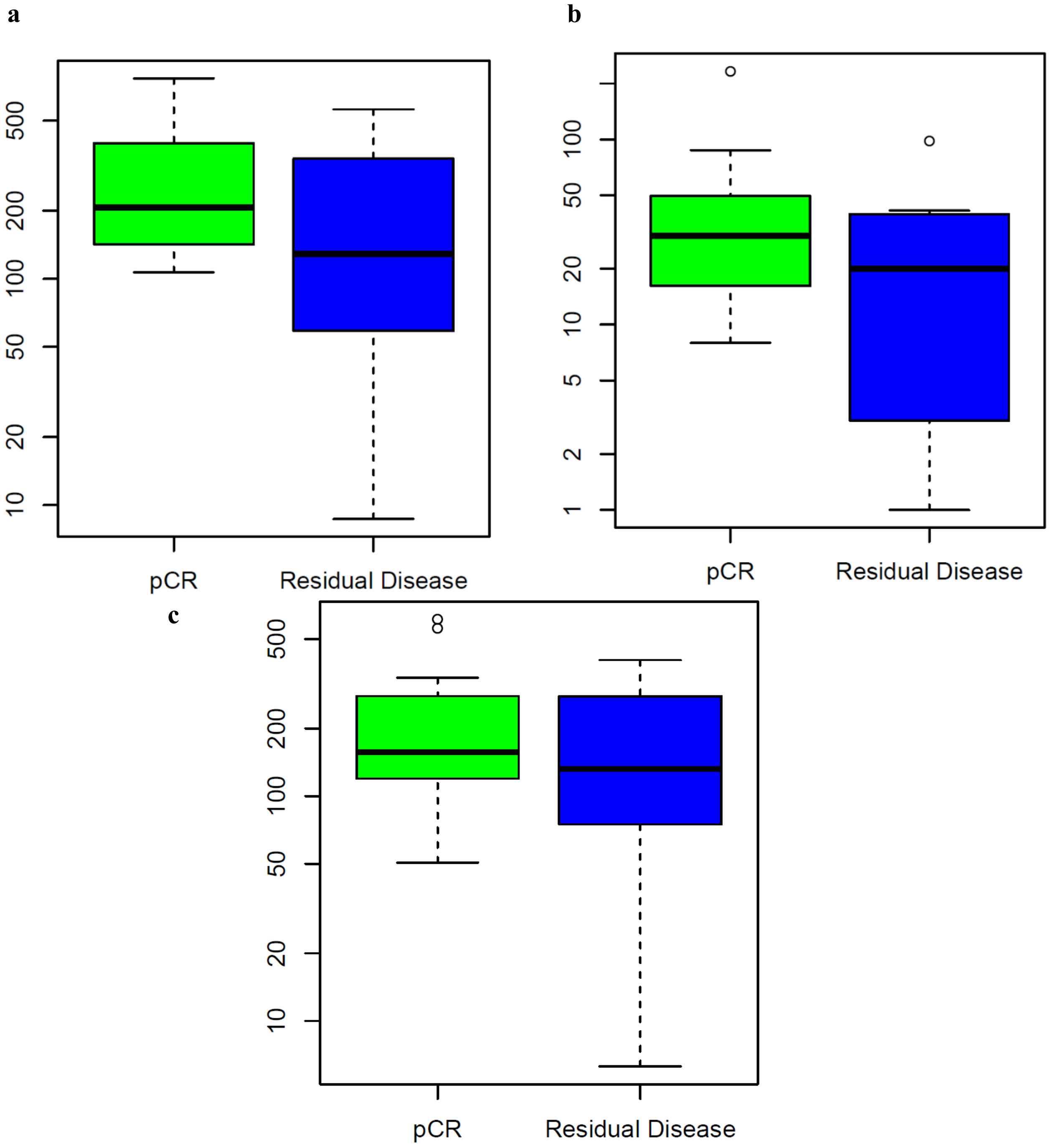

Supplementary Figure 1. Pathological complete response by multiplex immunofluorescence markers in patients treated with chemotherapy plus atezolizumab. Green areas represent pathological complete responses and blue areas represent non-pathological complete responses. (S1 A) Demonstrates CD8+ density cells/mm^2 in the chemotherapy plus atezolizumab, Arm B, p=0.25. (S1 B) CD8+PD1+ density cells/mm^2 in Arm B, p=0.33. (S1 C) FOXP3+ density cells/mm^2 in Arm B, p=0.48. The box stands for the interquartile range, the bar inside a box represents the median, and the whiskers represent 5th and 95th percentiles.

Supplementary Figure 2. Pathological complete response by menopausal status in patients treated with chemotherapy plus atezolizumab.

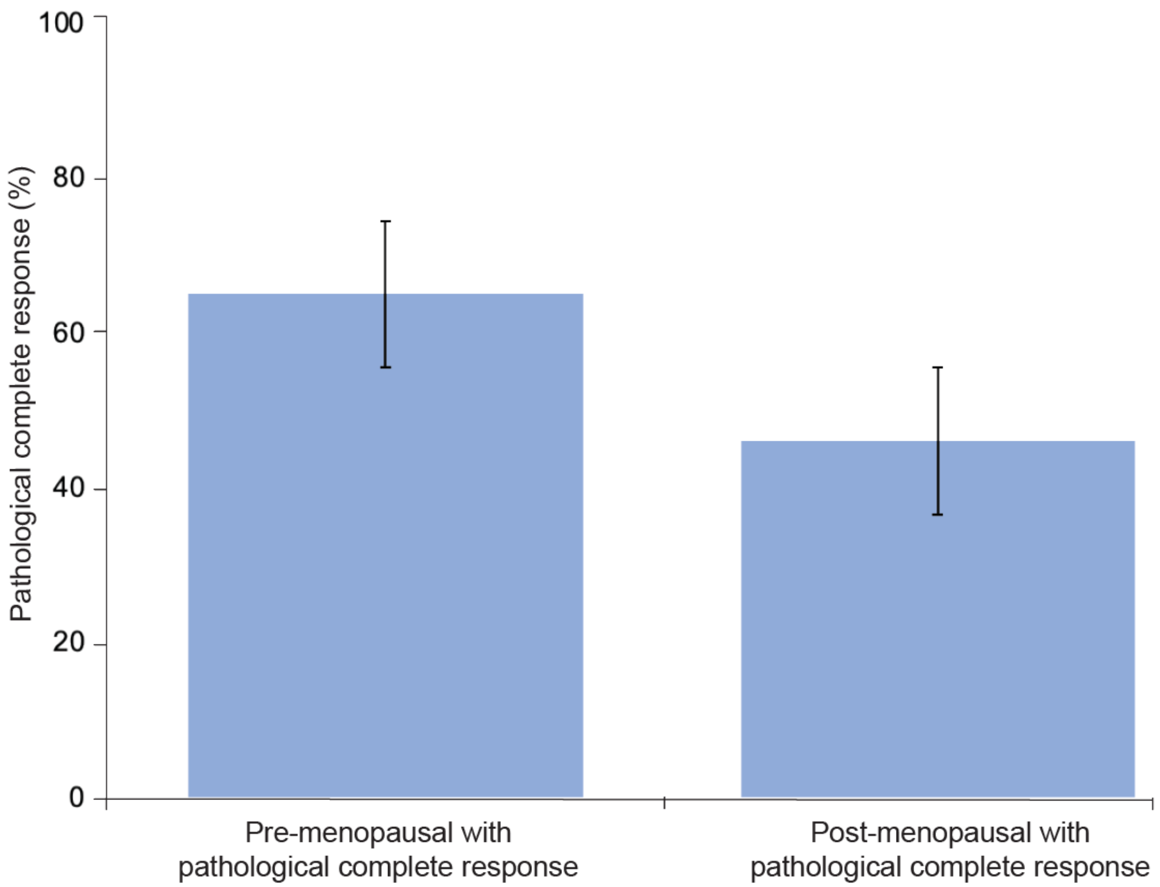

Supplementary Figure 2 Pathological complete response by menopausal status in patients treated with chemotherapy plus atezolizumab, p= 0.24. Error bars represent standard error of pCR rate.

Supplementary Figure 3. Pathological complete response by nodal status in patients treated with chemotherapy plus atezolizumab.

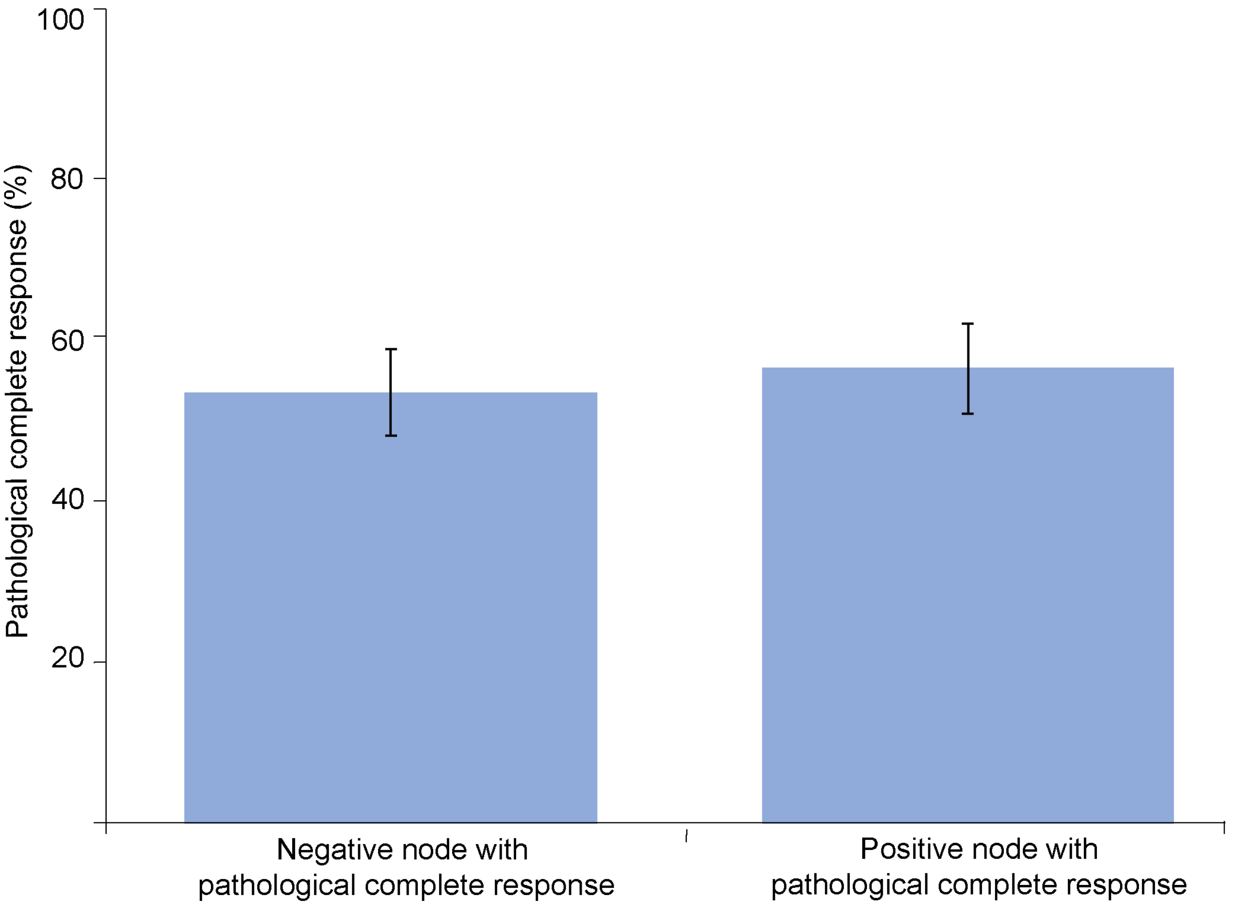

Supplementary Figure 3. Pathological complete response by nodal status in patients treated with chemotherapy plus atezolizumab, p> 0.99. Error bars represent standard error of pCR rate.
